# Cognitive Neuroscience Perspectives on Language Acquisition and Processing

**DOI:** 10.3390/brainsci13121613

**Published:** 2023-11-21

**Authors:** Yanina Prystauka, Vincent DeLuca, Alicia Luque, Toms Voits, Jason Rothman

**Affiliations:** 1 Department of Language and Culture, UiT the Arctic University of Norway, 9019 Tromsø, Norway; vincent.f.deluca@uit.no (V.D.); toms.voits@psy.gu.se (T.V.); jason.rothman@uit.no (J.R.); 2 Department of Foreign Languages and Translation, University of Agder, 4604 Kristiansand, Norway; 3 Nebrija Research Center in Cognition, Nebrija University, 28043 Madrid, Spain; aluque@nebrija.es; 4 Department of Applied Language Studies, Nebrija University, 28043 Madrid, Spain; 5 Department of Psychology, University of Gothenburg, SE-40530 Gothenburg, Sweden

The earliest investigations of the neural implementation of language started with examining patients with various types of disorders and underlying brain damage. The advent of neuroimaging tools in the twentieth century drastically changed the landscape of the field of the (cognitive) neuroscience of language, expanding the variety and depth of research questions one could ask without being confined to specific populations. Today we have better insights regarding the potential (neuro)cognitive correlates of language and an improved understanding of the neurocognitive consequences of language(s) in the mind/brain. And yet the linking hypotheses between neuroscience on the one hand and language on the other do not offer the level of detail needed to move the field from correlational to explanatory [1]. Thus, any further work that takes a more fine-grained look at both language processing and its neurocognitive substrates is warranted and welcome.

This Special Issue (SI) is dedicated to furthering our understanding of the neural processes underlying the dynamic nature of linguistic representations across development in the acquisition of language and during its real-time processing. We welcomed contributions that used cognitive neuroscience techniques and populations with diverse linguistic backgrounds, examining the relationship between language and other cognitive domains to extend our knowledge of language representation and computation in the brain.

The articles in this Special Issue cover a wide range of topics within the cognitive neuroscience of language; however, a few unifying themes emerge from this collection. Six of the nine articles that comprise the present SI investigate different aspects of sentence processing, specifically the processing of agent vs. patient intransitive subjects, case and adjective-noun order, relative clauses, figurative language, and temporal connectives. The neurocognitive components are examined either using neuroimaging techniques (EEG and fMRI) during sentence processing or testing diverse populations (individuals with aphasia and multilingual speakers). Processing is examined in Spanish, Latin, Mandarin, Russian, and English. Two of the nine articles examine the relationship between individual differences in language background and other aspects of domain-general cognition, specifically theory of mind (TOM) and episodic memory. Lastly, one paper investigates the electrophysiological and behavioral correlates of learning abstract and concrete words. In what follows, we will have a closer look at each of these papers, highlighting their contribution to our understanding of language in the mind and brain.

Two papers examine the processing of relative clauses. Akhavan and colleagues (contribution 1) investigate the effect of lexical-semantic cues during real-time sentence processing using the visual world paradigm (VWP) and eye-tracking in individuals with aphasia and neurotypical controls. Sentential materials in the study included object-relative clauses, where the meaning of the adjective in the main clause was manipulated such that it was either semantically related (biased adjective condition) or unrelated (unbiased adjective condition) to the displaced noun phrase, e.g., *The eagle saw the voracious/venomous snake that the bear cautiously encountered underneath the narrow bridge*. The authors hypothesized that the cues in the biased adjective condition would enhance lexical encoding and downstream syntactic retrieval for both groups. The results showed that the comparative group of neurotypical participants exhibited increased activation of relevant nouns and deactivation of irrelevant nouns in the presence of biased adjectives, resulting in improved retrieval of the target noun at the gap site. This aligns with previous research indicating that semantically richer noun phrases are more accessible during sentence processing [2,3,4,5,6]. However, the individuals with aphasia did not show sensitivity to these lexical-semantic cues in their initial lexical access. Instead, they demonstrated reduced interference from competitor nouns in the post-verb-frame window, indicating delayed but eventual processing of distinctiveness. This lack of sensitivity may be attributed to difficulties in accessing and maintaining representational features in real-time due to their aphasia-related impairments. These findings suggest that adding biasing adjectives as premodifiers may not be an effective strategy for individuals with aphasia to enhance representational access, given their initial delays in accessing features. Overall, this study sheds light on the complexities of lexical-semantic processing in aphasia and highlights the need for tailored approaches to support individuals with language impairments.

Also using VWP and eye-tracking, Stern and colleagues (contribution 2) investigate the role of language dominance to understand variability in the time course of first language (L1) processing of subject and object relative clauses among highly proficient Spanish-English bilingual speakers. In general, research shows a default preference for subject relative clauses over object relative clauses, presumably related to differences in the formal complexity of the two relative clause types leading to differential processing loads implicated for each and agency over patienthood [7,8,9]. The study replicated these two general tendencies. First, participants exhibited a semantically driven preference to assign the thematic role of agent to the referents of lexically animate noun phrases (NPs). Second, there was a syntactically driven preference in play, particularly when it came to interpreting relative clauses. Participants leaned toward subject-extracted relative clauses (SRCs) over object-extracted clauses (ORCs). The influence of language dominance emerged as a compelling aspect of this study. Individuals with greater comfort and proficiency in one language over the other were less inclined to favor the agency default. Rather, they were more open to embracing the specific sentence structures linked to SRCs. This intriguing relationship between preferences and language dominance is not one-dimensional. Instead, it seems to originate from separate cognitive mechanisms, rendering it a complex phenomenon. The study proposed several hypotheses to explain these findings. It was speculated that linguistic characteristics specific to the participants’ languages, notably Spanish as their first language, may play a role. The structure of Spanish sentences, particularly those emphasizing affected entities over agents, could contribute to this phenomenon. Alternatively, the study suggested a potential trade-off between processing strategies, wherein a heightened reliance on syntactic structures accompanies a reduced emphasis on semantic agency preferences as language dominance increases. This proposal aligns with the idea that language-specific factors play a relevant role in shaping individuals’ language-processing strategies. Furthermore, the study raised the possibility that individual cognitive skills, such as cognitive control and working memory, could influence the relationship between semantic and syntactic processing strategies, suggesting that these individual cognitive factors may play a pivotal role in how individuals navigate competing cues during language comprehension. In conclusion, this study sheds light on the interplay between language dominance, cognitive processes, and linguistic preferences as underlying mechanisms that might help explain the dynamic nature of language processing in diverse bilingual contexts. An interesting further direction in this present space would be to test individuals with aphasia (monolinguals and bilinguals alike) on the paradigm reported in Stern et al. (contribution 2) and bi-/multilingual individuals on the paradigm from Akhavan et al. (contribution 1) to further examine how linguistic background affects reliance on semantic and syntactic information.

Another study that used bilingualism as a window into neural plasticity was by Zawiszewski and colleagues (contribution 3), who explored the longstanding question of whether non-native speakers can process their second language (L2) in a native-like way [10,11,12,13,14]. The study focuses on comparisons in processing grammatical and ungrammatical subject-verb agreement dependencies, particularly on agent and patient subject predicates and person and number phi-features across different speaker groups. Monolingual Spanish native speakers and early Basque-Spanish bilinguals highly proficient in Spanish participated in the study. Participants completed a grammatical judgment task while the EEG was recorded, involving sentences with varying agent and patient subject predicates and person and number phi-features. Behaviorally, while Basque-Spanish bilinguals demonstrated high proficiency in Spanish, they were marginally slower and less accurate in judging grammatical sentences than the Spanish functional monolingual speakers. These discrepancies were attributed not to differences in competence but possibly to a more moderated automaticity in deploying processing resources for bilingual speakers. Neurophysiologically, a pattern emerged with all participants exhibiting left-lateralized negativity toward number violations, highlighting the easier distinction between number and person violations. Both groups perceived patient-subject predicates as more challenging to process than agent-subject predicates, aligning with the agent-first hypothesis [15], whereby patient-subject sentences incur greater processing costs due to initial assumptions about sentence-initial non-marked animate arguments being agents. However, in the 300–500 ms window, L1 Spanish dominant native speakers displayed heightened sensitivity to distinctions between patient and agent subject predicates compared to Basque-Spanish bilinguals, the latter showing smaller effects with subject-verb agreement violations, likely due to reliance on case morphology present in their L1, Basque, but absent in Spanish. These processing differences align with the Language Distance Hypothesis (LDH) [16], suggesting that disparities in processing between functional monolinguals and bilingual speakers may stem from differing grammatical phenomena in the L1 and L2 rather than from variations in linguistic competence. In conclusion, this study reaffirms the agent-first hypothesis, underscoring that for monolingual and bilingual speakers, sentences with patient subjects impose greater processing costs than those with agent subjects due to an initial presumption that all sentence-initial non-marked animate arguments are agents. It also lends support for accounts suggesting differing processing mechanisms for person and number features and highlights the premise that, with early acquisition and at high(er) levels of proficiency, target processing can be attained beyond an L1, provided that linguistic properties are congruent between the L1 and the L2.

Congruency of structural properties among languages is also argued to be a crucial predictive factor in additive multilingual (third or more language (L3/Ln)) acquisition and processing [17,18,19,20]. Models on L3 acquisition differ with respect to how they envisage the degree (holistic vs. selective transfer of the L1, L2, or both) and/or timing (initial stages vs. development) of how the influence of source languages unfolds [21,22,23]. With this theoretical landscape in mind, Pereira Soares and colleagues (contribution 4) used EEG/ERPs herein to examine these models, bringing together two types of bilinguals: Italian-German heritage speakers and adult German native speakers L2 learners of English. Following Rothman et al. [24] and González Alonso et al. [25], they used a mini-grammar learning paradigm to control the quantity and quality (the degree of overlap with the other languages previously acquired) of input, ensuring that the participants were at the true initial stages of L3/Ln development, and used an EEG paradigm to examine what, if any, neural signatures might reveal with respect to previous language transfer at the very initial stages. Accordingly, participants were trained on a selected Latin lexicon over two sessions (allowing for a consolidation period) and, afterward (i.e., in session 2), on two grammatical properties: case (similar between German and Latin) and adjective-noun order (similar between Italian and Latin). Neurophysiological findings show an N200/N400 deflection for the heritage speakers in case morphology and a P600 effect for the German L2 group in adjectival position, the former indicating differential attention allocation being recruited as a function of this group’s being early bilinguals, which the authors suggest makes them more sensitive to morphological contrasts in general (see also [26,27]). The latter, the P600 effect, conversely indicates that learning the target property within the experimental paradigm must be explanatory, given that Germany could not have provided transfer for this property. As a result, none of the current L3 models straightforwardly account for the observed results in the sense that they do not predict the observed performances. The authors give a set of well-grounded reasons as to why they ultimately question the appropriateness of the methodology in its current formulation for L3/Ln theory testing, making suggestions for how the method can be modified to do so in the future. Nevertheless, the results are illustrative of differences in how HSs and L2 learners approach the very initial stages of additional language learning itself.

While these first three studies compared groups of participants with different characteristics, an emerging trend in the field (which is also reflected in our special issue) is the increasing interest in individual differences. The following cohort of studies takes advantage of this approach. The study by Yin and Yang (contribution 5) is a functional neuroimaging investigation of the neural processing of metaphor and metonymy in bilingual individuals. Metaphor and metonymy are types of figurative language with the core difference that metaphor allows for explaining one thing in terms of another (e.g., knowledge is power, time is a thief), whereas metonymy refers to a thing by using a particular property of some other thing (e.g., the pen is mightier than the sword, the ballot is stronger than a bullet). More specifically, the authors studied the moderating effects of working memory capacity and vocabulary size on the neural correlates of metaphor and metonymy computation in L2 English in a sample of Mandarin-English bilinguals. In a novel departure from prior literature (e.g., [28,29,30]), the authors differentiate between multiple subtypes of metonymy, namely systematic (requiring increased contextual demands) and circumstantial (more context-specific) metonymy. The rationale behind this distinction is the belief that different types of metonymy may impose variable computational demands. The participants completed a set of language background questionnaires and a valence judgment task involving literal, metaphoric, and metonymic contexts while undergoing an MRI scan. Behaviorally, the participants displayed similar response times irrespective of language type (literal, metaphoric, or metonymic). However, follow-up analyses revealed a trending effect of subtype (systematic, circumstantial), with reaction times slower in the systematic subtype trials. Regarding the neural substrate of figurative language processing, Yin and Yang replicate previous findings highlighting activations in the fronto-temporal networks during figurative language processing [31]. Additionally, they show a differential involvement of the right supramarginal gyrus, right cerebellum, and left precentral gyrus in metonymy processing compared to metaphor processing and report neurofunctional effects of vocabulary size and working memory capacity specifically in circumstantial metonymy processing, but not metaphor nor systematic metonymy. This study underscores the importance of considering individual differences in neurolinguistic research on figurative language processing.

The study by Chen and colleagues (contribution 6) examined the degree to which temporal connectives affect the retrieval and integration of world knowledge information in language/sentence processing (and its neural underpinnings) in Mandarin. The study builds off previous work that shows the temporal connectives “before” and “after” differentially affect the integration of world knowledge into sentence processing and the neural correlates to this, with “before” sentences showing greater difficulties in processing integration than sentences with “after”, in line with an iconicity account that describes that sentences are easier to process if the sequencing of events matches the sequencing of clauses [32,33]. Chen et al.’s study extends this examination to Mandarin, where temporal connectives are always in a consistent sentence position, making it easier to examine accounts of iconicity in processing. A cohort of young adult speakers of Mandarin (*n* = 32, mean age: 21.8 years, SD: 2.92) completed a sentence truth-value judgment task with temporal connectives and a critical word following these that were congruent or incongruent with participants’ world knowledge (based on several prior cloze tests and forced-choice judgment tasks), while EEG was recorded. ERPs were calculated, locked to the onset of the critical (congruent/incongruent) word. Behavioral results showed no significant effects on sentence types (although a trending effect is reported where incongruent sentences were slightly more difficult to comprehend than congruent sentences). The ERP result showed that incongruent sentences with “after” showed a significantly stronger P600 effect than congruent sentences with “after”, which was not found for the “before” sentences. However, “before”-congruent sentences showed stronger late positivities than “after”-congruent sentences. Moreover, a follow-up analysis of the sentence-final word showed that incongruent sentences elicited greater negativities between 300 and 800 ms than congruent sentences. Finally, the authors also report a correlation between P600 responses and working memory in one direction, which suggests that participants with higher working memory capacity are more efficient in processing the reverse temporal relations.

Switching gears, a final set of two articles discuss not language processing aspects per se but rather the interaction of language backgrounds with other domain-general functions such as the theory of mind (TOM) and episodic memory. TOM refers to our ability to understand the intentions, beliefs, and knowledge of others based on their behavior [34,35,36,37], whereas episodic memory refers to a distinct neurocognitive system allowing humans to remember past experiences [38,39]. The article by Navarro et al. (contribution 7) assesses the effects of individual differences in bilingual experience on TOM outcomes in young adults. Navarro and colleagues specifically examine how the effects of the broader sociolinguistic factors affect TOM performance, including self-driven (ego) experiences, one’s experiences with personal contacts (ego-alter), and the respective relationships (in terms of language use) of one’s social environment (alter-alter). A sample of young adults with diverse (bilingual) language experience was recruited and underwent a battery of measures, including the director task (measure of TOM), metalinguistic awareness (based on [40]), and a social network index that measured individuals’ personal language experiences and those of their immediate social environment. Measures from the social network questionnaire were aggregated into constructs (e.g., ego switching, alter-alter language use), which were subsequently regressed against task performance in the director task and metalinguistic awareness task. Performance on the director task significantly correlated with (1) degree of second language (L2) use, (2) frequency of switching, and (3) degree of engagement with both languages by alters in childhood. These data show diversity in language exposure in one’s broader sociolinguistic environment also has implications for neurocognitive adaptations, herein seen as TOM performance. The results, furthermore, add to a growing body of literature showing neurocognitive outcomes are calibrated to the degree of bilingual experience across the lifespan.

While Navarro et al. (contribution 7) examined the effects of diversity on participants’ current and previous experience with language, including their family networks’ experience with language, Antón and Duñabeitia (contribution 8) explored the effects of language mixing during the presentation of biographical information on retention of this information within a large cohort of bilingual Basque-Spanish children. The participants were presented with cartoon-like 3D avatars, each characterized by their own set of biographical features. The information was distinct for each avatar, and so were their voices. Notably, the avatar-language association was manipulated, with some avatars speaking Spanish, some Basque, and some intersententially code-switching between the two languages (*mixed* condition). Participant memory was assessed through a series of free recall and recognition questions immediately following the exposure phase and also a day later. The authors reported a significant main effect of self-reported Basque proficiency, with higher proficiency associated with greater accuracy in recall and recognition. Moreover, two interactions were significant, showing that (1) accuracy increases with age on the immediate recall and recognition tests while remaining constant across age in the delayed test, and (2) accuracy remains stable for Basque and mixed conditions across ages but drops with age for the Spanish condition. Crucially, no evidence was found for diminished memory performance as a function of mixed-language context. This finding has profound implications for education and schooling policies.

Since individual differences (for example, the age of acquisition of a particular word/structure) are not always included as a factor/controlled for, Mkrtychian and colleagues (contribution 9) focused on both the behavioral and neural (EEG) aspects of the acquisition and processing of concrete and abstract words while tightly controlling for other potential confounds that have been overlooked in previous work, e.g., psycholinguistic properties (frequency, length, etc.), age of acquisition, acquisition mode, experimental techniques, etc. To achieve this, the researchers used a controlled experimental design where participants learned novel concrete and abstract words in a sentence context. To test word acquisition, the authors utilized a few behavioral tasks tapping into the lexical and semantic aspects of the newly learned words as well as recording participants’ EEG in a reading task. Behaviorally, both word types were successfully learned after only five presentations; however, there were differences between different tasks. Participants performed numerically but not significantly better on concrete words in semantic tasks. Conversely, abstract words showed advantages in lexical tasks, with higher accuracy and faster reaction times, contradicting traditional concreteness effects. EEG results revealed differences in brain activation between concrete and abstract words. Concrete words elicited a stronger response at around 146 ms relative to abstract and control untrained pseudowords, while abstract words only showed a difference relative to control pseudowords at 206 ms. These early neural differences contrast with previous research that primarily identified differences in later components of word processing (such as N400 and N700). The topographies and results of source reconstruction align with prior research, indicating that, despite some variations in the timing of effects, the general localization of newly acquired abstract and concrete concepts is quite similar. The source localization underscores the significance of Wernicke’s area and its right hemisphere counterpart in the process of word acquisition. These findings are consistent with previous studies showing that both brain hemispheres play a role in acquiring abstract and concrete words. The study’s results have implications for our understanding of word acquisition and semantic processing. However, there are limitations to consider, such as the need for future research to examine the long-term effects of word acquisition, explore acquisition modalities, and verify findings in other languages and populations. Additionally, the study opens avenues for further investigations into different subtypes of semantics. 

In conclusion, the articles in this special issue have provided valuable insights into the complex interplay between language, cognition, and the brain. One overarching theme that emerges from these articles is the significance of individual differences. Whether it is the impact of language dominance on syntactic preferences, the role of working memory in processing temporal connectives, or the influence of bilingual experience on the theory of mind, these studies highlight the importance of considering the unique characteristics of each individual when studying language and cognition. As we continue to explore these complex relationships in combination with fine-grained linguistic theories, smart experimental techniques, and advanced neuroimaging techniques, we can look forward to further advances in our understanding of language in the mind and brain.
